# Temperature Evolution of Two-State Lasing in Microdisk Lasers with InAs/InGaAs Quantum Dots

**DOI:** 10.3390/nano13050877

**Published:** 2023-02-26

**Authors:** Ivan Makhov, Konstantin Ivanov, Eduard Moiseev, Nikita Fominykh, Anna Dragunova, Natalia Kryzhanovskaya, Alexey Zhukov

**Affiliations:** International Laboratory of Quantum Optoelectronics, HSE University, Soyuza Pechatnikov Str., 16, St. Petersburg 190008, Russia

**Keywords:** two-state lasing, quantum dots, microdisks, ground-state, excited-state, temperature, whispering gallery modes

## Abstract

One-state and two-state lasing is investigated experimentally and through numerical simulation as a function of temperature in microdisk lasers with Stranski–Krastanow InAs/InGaAs/GaAs quantum dots. Near room temperature, the temperature-induced increment of the ground-state threshold current density is relatively weak and can be described by a characteristic temperature of about 150 K. At elevated temperatures, a faster (super-exponential) increase in the threshold current density is observed. Meanwhile, the current density corresponding to the onset of two-state lasing was found to decrease with increasing temperature, so that the interval of current density of pure one-state lasing becomes narrower with the temperature increase. Above a certain critical temperature, ground-state lasing completely disappears. This critical temperature drops from 107 to 37 °C as the microdisk diameter decreases from 28 to 20 μm. In microdisks with a diameter of 9 μm, a temperature-induced jump in the lasing wavelength from the first excited-state to second excited-state optical transition is observed. A model describing the system of rate equations and free carrier absorption dependent on the reservoir population provides a satisfactory agreement with experimental results. The temperature and threshold current corresponding to the quenching of ground-state lasing can be well approximated by linear functions of saturated gain and output loss.

## 1. Introduction

Modern tendencies in optical communications are associated with the ever-growing volumes of transmitted information, leading to a requirement to increase data transfer rates and throughput of the optoelectronic systems, as well as to increase the performance of the devices, coupled with a reduction in their sizes. In this case, the use of photonic integrated circuits [1,2] is one of the possible ways to complete the abovementioned tasks. Microlasers based on whispering gallery modes [3,4] are one of the promising light sources for the implementation with photonic integrated circuits. Such microlasers supplemented with a III–V quantum dot (QD) active region demonstrate operating characteristics of low-threshold currents and, good thermal stability, together with a high-speed modulation and low lateral dimensions [5,6,7,8]. It should also be noted that QD-based microlasers can be monolithically grown on silicon [9,10,11], as well as transferred to silicon from the native substrate [12,13] without performance degradation [14]. 

Quantum dots used as the active region of lasers demonstrate gain saturation [15], which primarily depends on the density and homogeneous broadening of QD states. In short, the maximum gain on the ground-state (GS) optical transition of QDs is achieved if the QDs are completely populated with two electrons and two holes on the corresponding GS levels. The gain saturation, along with finite carrier relaxation times between QD states, can result in a two-state lasing associated with the emergence of simultaneous lasing via different optical transitions of QDs (for example, ground-state and first excited-state optical transitions) [16,17]. In this case, the spectral distance between two lasing lines can be up to 70–100 nm, depending on the energy distance between different optical transitions of QDs. 

On the one hand, existence of numerous exited-state optical transitions and saturation of the gain at the GS optical transition of QDs can be considered negative effects, since they make it difficult to achieve lasing in a narrow spectral range when the operating conditions of the laser (temperature, injection current) or its design (output loss) are changed. On the other hand, excited-state lasing or two-state lasing phenomenon may have several possible applications. One of these is related to the development of the laser sources with an extremely broadband emission spectrum (up to 75 nm to date) with a high output power, which can be achieved via intentional inhomogeneous broadening of the quantum dot states and involvement of the ground and first-excited optical transitions of quantum dots [18,19]. Another is the realization of wide and flat optical gain spectra (also in combination with chirping the QD planes) for broadly tunable lasing with a continuous tuning range over 200 nm [20]. Yet another is related to the use of two-state lasing for the realization of spectral coding related to the data transmission on several wavelengths corresponded to the different optical transitions of quantum dots [21]. The use of spectral coding should result in increased throughput of the data transmission systems, due to the transition from two- to four-level modulation [22]. It should also be noted that switching of lasing between different transition in quantum dots in a two-state regime can be fast on the order of 100 ps [23,24,25], leading to the achievement of high-speed modulation. 

Such a spectral coding can be also realized for the data transmission in photonic integrated circuits in order to increase their performance. For this purpose, lasers of miniature size, e.g., QD microdisk lasers, are attractive. To date, the two-state lasing regime was already observed in injection microdisk lasers in continuous-wave regime at room temperature [26]. The possibility to achieve two-state lasing via ground and first-excited, as well as first- and second-excited optical transitions in quantum dots, was also demonstrated for microdisk lasers [27]. During the operation, the temperature of microlasers can be elevated due to the laser self-heating [28] or due to heating from the neighboring elements, leading to the change in loss and in distribution of charge carriers between states of quantum dots. As a result, the two-state lasing conditions should significantly depend on the temperature of the microlaser. Taking into consideration the above, the present work is devoted to an experimental and theoretical studies of the temperature evolution of two-state lasing characteristics in InAs/InGaAs QD microlasers with disk cavities of various diameters.

## 2. Materials and Methods

The experimental studies were carried out using a molecular beam epitaxy grown laser heterostructure with QD active region. On an *n*^+^-GaAs substrate, a 500 nm-thick *n*^+^-GaAs buffer layer was grown, followed by an *n*^+^-Al_0.25_Ga_0.75_As cladding layer of 2.5 μm thickness. A further 10 layers of QDs formed by consistent deposition of 2.5 monolayers of InAs, followed by deposition of 5 nm In_0.15_Ga_0.85_As, were grown. Each quantum dots layer was separated from each other with a GaAs spacer of 35 nm thickness. The central 10 nm of the GaAs spacer was doped with a *p*-type impurity at a level of 5·10^17^ cm^−3^. The QDs were located in the middle of a 0.4 μm-thick GaAs waveguide. Then, a *p*^+^-Al_0.25_Ga_0.75_As cladding layer of 2.2 μm thickness was grown, followed by a 200 nm-thick *p*^+^-GaAs contact layer.

**Figure 1:**
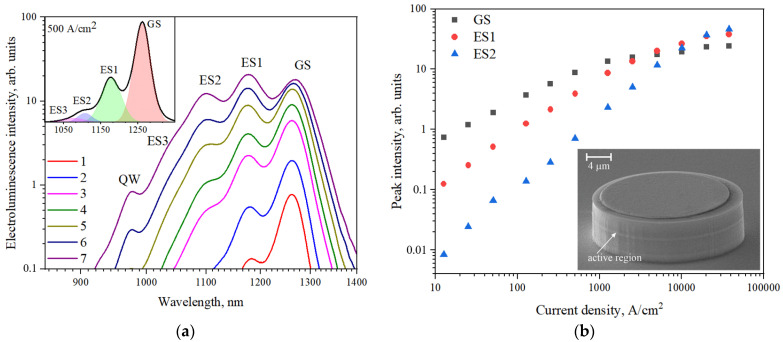
Spontaneous electroluminescence spectra (**a**) of the heterostructure with InAs/InGaAs quantum dots, measured at the room temperature at different injection currents (1: 15 A/cm^2^; 2: 50 A/cm^2^; 3: 250 A/cm^2^; 4: 500 A/cm^2^; 5: 1250 A/cm^2^; 6: 2500 A/cm^2^; 7: 5000 A/cm^2^). The inset shows the decomposition of the electroluminescence spectrum (4) with the Gauss contours; (**b**) peak intensities of three optical transitions against current density. Inset: microscopic image of the 24 μm microdisk.

The photolithography and plasma-chemical etching were used to form microdisk cavities with outer diameters of 28, 24, 20 and 9 μm. The height of the formed microcavities was about 6 μm. Each microlaser had a round-shaped metal *p*-contact made of AgMn/Ni/Au metallization at the top of the microcavity for the connection to the *p*^+^-GaAs layer. A solid AuGe/Ni/Au metallization was deposited to the back side of the preliminarily thinned substrate used as *n*-contact. The scanning electron microscopy image of the 24 μm microdisk laser is presented in the inset to Figure 1b.

In order to realize good thermal stability of microlasers, chips with several microdisks were soldered with indium to a copper heatsink, supplemented with a built-in resistive heater, temperature sensor and PID-temperature controller, making it possible to investigate the temperature evolution of microlasers characteristics. In this case, the copper heat-sink had an electrical connection to the *n*-contact of the microlasers. Electrical connection to a *p*-contact of individual microlasers was attained with a BeCu microprobe supplemented with a 3-axis manual translation stage.

Although we did not focus on determining the maximum optical power of the devices under study in the present work, it should be mentioned that typical values reach 1 mW in continuous-wave regime. Aiming to diminish the microlaser self-heating, the bias voltage to the microlasers was applied in a pulsed regime with a pulse duration of 300 ns and repetition rate of 4 kHz. The amplitude of pulsed current was measured using an inductive probe based on a Rogowski coil and an oscilloscope Tektronix MDO34. Electroluminescence from the single microlaser was collected in the lateral direction with a microobjective (Mitutoyo M Plan APO NIR HR50x) and directed via a multimode optical fiber to the entrance slit of an Andor Shamrock 500i grating monochromator, supplemented with a diffraction grating containing 1200 lines per millimeter. A thermoelectrically cooled InGaAs CCD array was used as a photodetector. Spontaneous electroluminescence spectra were measured from the facet of the test structure formed from the same epitaxial wafer. Due to its shape (in the plane) of a triangular mesa, it is characterized by a low Q-factor, which does not allow lasing up to high injection currents.

## 3. Results

### 3.1. Spontaneous Emission of the Active Region

At the first stage of the experimental studies, the spontaneous electroluminescence spectra of the laser heterostructure without cavity-related effects were investigated at room temperature in order to obtain spectral positions of emission bands related to the ground-state and excited-state optical transitions in quantum dots. The obtained spontaneous electroluminescence spectra, measured at different current densities, are presented in Figure 1a. Several emission bands assigned to the ground-state (GS) and the first-, second- and third excited-state (ES1, ES2 and ES3, respectively) optical transitions are revealed in the emission spectra. In order to better clarify the contribution of these emission bands, the decomposition of the spectrum measured at the current density of 500 A/cm^2^ with Gauss contours is shown in the inset to Figure 1a. The observed spectral positions of the emission bands related to the ground and excited optical transitions in InAs/InGaAs quantum dots (GS: 1262 nm, ES1: 1175 nm, ES2: 1105 nm) are in agreement with the data obtained for similar QD structures [29,30]. 

At the highest injection currents, still another excited-state optical transition (ES3) manifests itself as a weakly pronounced change in the slope of the spectral curve near 1025 nm. In addition, the emission band, which is attributed to the radiative electron-hole recombination in the covering InGaAs quantum well, is observed close to the wavelength of 985 nm. The intensities of the optical transition peaks are presented in Figure 1b as a function of the injection current. One can see that at, low injection currents, the ground-state luminescence band dominates in the emission spectrum. The current increase leads to the almost consistent emergence of the emission bands associated with the excited-state optical transitions. Starting from approximately 1 kA/cm^2^ injection current density, the saturation of the GS emission band intensity becomes apparent, which can be explained by nearly full occupation of the ground-state level with charge carriers. Further, the intensity of the emission bands associated with several excited-state optical transitions continue growing almost linearly with the current density, because of incomplete population of those excited-states levels having higher degeneracy factors compared to the ground-state one. Starting from 5 kA/cm^2^, the intensity of the ES1 band exceeds that of the GS band. At 20 kA/cm^2^, the ES2 emission peak becomes dominant. The observed evolution of the spontaneous emission of quantum dots with the pumping power was also observed by other research groups [31,32].

### 3.2. Two-State Lasing in Microdisk Lasers

**Figure 2:**
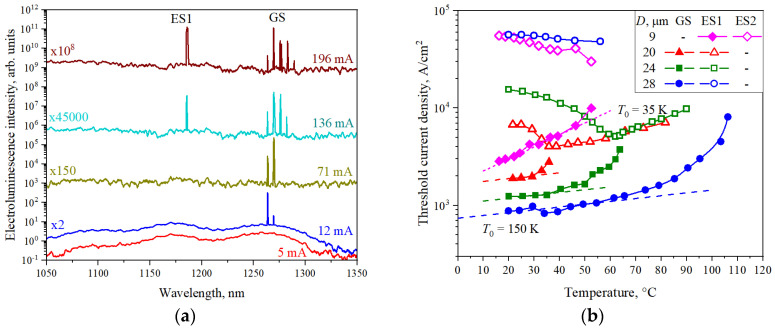
(**a**) Electroluminescence spectra of the microlaser with disk diameter of 24 μm measured at the different injection currents at room temperature; (**b**) Temperature evolution of the threshold current densities of the first lasing (solid symbols) and two-state lasing (open symbols) for GS, ES1 and ES2 optical transitions in microdisks of different diameters (28 μm: circles, 24 μm: squares, 20 μm: triangles, 9 μm: rhombs). Dashed curves correspond to the characteristic temperature of 150 K and dotted lines correspond to 35 K.

At the next stage of the studies, the emission from the microdisk lasers was investigated. Figure 2a shows the room temperature electroluminescence spectra of the 24 μm microlaser at different injection currents. It is seen that, at low injection current (5 mA), the microlaser demonstrates the same spontaneous emission bands related to the ground-state and excited-state optical transitions of quantum dots as the sample without cavity discussed above. The current increase leads to the emergence of the laser emission. Several narrow lines are located within the GS band of emission (around 1265 nm). They are separated from each other by 5.5 nm, which corresponds to the free spectral range expected for whispering gallery modes in a GaAs microdisk of the corresponding diameter. As injection current reaches > 100 mA, the second group of lasing line arises in the emission spectrum at the wavelength of about 1185 nm. This wavelength corresponds to the ES1 optical transition of quantum dots. Thus, we can state that, at sufficiently high injection currents, two-state lasing is observed in QD microdisk lasers, similar to what occurs in edge-emitting lasers with QDs. Further increase of pumping currents results in the intensity increase for the ES1 lasing and appropriate decrease in the GS lasing intensity.

In the investigated microdisks with cavity diameters of 20–28 μm, two-state lasing involves the GS and ES1 optical transitions. Herewith, a decrease in the cavity diameter results in the increase in the first (GS) lasing threshold, along with simultaneous decrease in the second (ES1) lasing threshold. This indicates that the optical loss increases with decreasing microdisk diameter. In the 9 μm microdisk, no lasing via the GS optical transition is observed, and the first lasing line emerges already on the ES1 optical transition. This can be explained by the fact that the optical loss in disks of such a small diameter exceeds the saturation gain for the GS optical transition. In case of the 9 μm microlaser, the two-state lasing occurs via the ES1 and ES2 optical transitions of quantum dots. A more thorough investigation of two-state lasing in microdisk lasers of different diameters with InAs/InGaAs quantum dot active region at room temperature is presented in [27]. A comparison of two-state lasing behavior of the microdisk lasers and the stripe broad-area lasers made of the same epitaxial wafer allows us to conclude that optical loss in the microdisks under study is inversely proportional to their diameter, with a proportionality coefficient of about 421 cm^−1^×μm.

### 3.3. Temperature Evolution of the Two-State Lasing: An Experimental Study

As was mentioned above, the main purpose of the present work is to study the characteristics of the two-state lasing regime at different temperatures in view of the prospects for the operation of QD microdisk lasers at elevated temperatures. Figure 2b shows the obtained temperature dependences of the threshold current densities for microlasers of different disk cavity diameters. As can be seen, the threshold current density of GS lasing, measured in 28-, 24-, 20-μm microdisks, increases with temperature. Near room temperature, the temperature-induced increment of the threshold current density is relatively weak and can be described by a characteristic temperature *T*_0_ of about 150 K for all three diameters. This means that the temperature dependence of the threshold current density $J_{th}^{GS}$ is determined by processes that are the same for all diameters, i.e., independent or weakly dependent on loss. We believe that, in this temperature interval, the growth of the threshold current density is caused by temperature-activated nonradiative recombination and faster carrier escape out of the ground-state level of QDs to higher-energy states. The rate of the later process increases exponentially with temperature [17], so that a higher injection current is required to achieve the same (threshold) occupation of the GS level at an elevated temperature. Simultaneously, the ES1 level becomes more populated with charge carriers, which explains the decrease in the threshold current density of two-state lasing.

**Figure 3:**
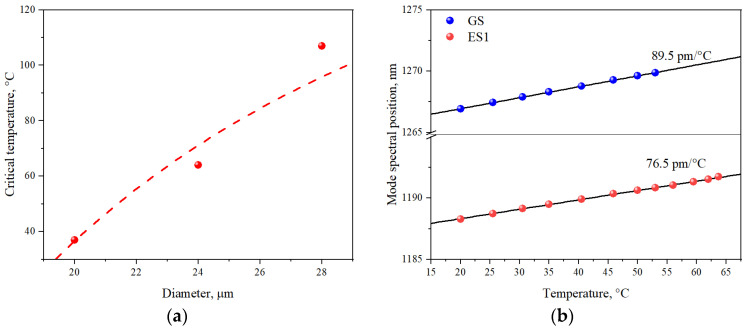
(**a**) Critical temperature of GS lasing against microdisk diameter: experiment (symbols) and simulation (dashed line); (**b**) Dependence of the dominant lasing mode spectral position on the temperature, obtained close to the lasing threshold for the GS and ES1 lasing. Solid lines correspond to the linear dependences.

There is also a temperature-induced growth of the carrier concentration in the waveguiding layer. This should lead to an increase in optical loss by free carrier absorption [33,34] and, therefore, to a more rapid rise of the threshold current density. It can be seen that the temperature, which corresponds to the beginning of a faster (super-exponential) increase in the threshold current, shifts towards lower values as the microdisk diameter decreases. For example, *T*_0_ of 150 K satisfactorily describes the temperature behavior of $J_{th}^{GS}$ up to approximately 60 °C for the 28 μm microdisk, while it is only 30 °C for the 20 μm microdisk. Since the threshold current density of GS lasing increases and the threshold current density of two-state lasing decreases with increasing temperature, the current density interval of pure GS lasing becomes narrower. Above a certain critical temperature, $T_{cr}$, GS lasing disappears. In the microdisk laser with a diameter of 28 μm, the critical temperature is 107 °C. It is worth mentioning that, in the edge-emitting laser with the stripe length of 1 mm, GS lasing disappears at the same temperature of 107 °C. As shown in Figure 3a, the critical temperature is found to be dependent on the microdisk diameter (i.e., on the optical loss) and decreases to 37 °C and 64 °C for the diameters of 20 and 24 μm, respectively. 

For the 9 μm microdisk, where lasing starts through the ES1 optical transition, the characteristic temperature of the threshold current is low: only 35 K. Otherwise, the temperature behavior for this smallest microdisk is similar to that discussed for larger lasers. Again, a decrease in the current density corresponding to the two-state lasing threshold (through ES2) is observed up to the complete cessation of emission through the first excited-state transition at the critical temperature. In this case, it is about 55 °C.

It should also be noted that temperature increase also lead to the redshift of lasing whispering gallery modes. Figure 3b shows spectral positions of the main modes for the GS and ES1 lasing obtained for the 24 μm microdisk close to the threshold currents at different temperatures. One can see that GS and ES1 lasing modes shift to the longwave region, with coefficients of about 89.5 and 76.5 pm/°C, respectively. The obtained values of these coefficients are in good agreement with the values determined for similar microlasers with quantum dot active region [35]. The general reason of such temperature-induced shifts of lasing mode are associated with the change of the effective refractive index of the active region, as well as with the change in the linear thermal expansion coefficient with the temperature rise [36]. Slight difference in the observed mode shift coefficients for the GS and ES1 lasing can be attributed, in addition to bare effect of different central wavelength, the presence of spectral dependence for the refractive index. 

### 3.4. Temperature Evolution of Two-State Lasing: Numerical Modelling

Aiming to theoretically describe the two-state lasing phenomenon in the investigated microdisk lasers with quantum dots, numerical simulation was conducted. Numerical modelling was carried out using a system of rate equations for populations of the ground level ($f_{1}$), three excited levels ($f_{2}$–$f_{4}$) and the reservoir $n_{res}$ in accordance with the results obtained from the spectral studies of the spontaneous electroluminescence of quantum dot active region described above (see Figure 1a and its discussion in Section 3.1), as well as for corresponding photon modes $s_{1}$–$s_{4}$. The equations are similar to those described in [37,38], where a similar two-level system was considered. We extended the model in order to include into consideration higher excited energy levels and, in particular, the second excited-state level of QDs, which is involved in lasing of the smallest microdisks. The modified rate equations are as follows:

(1) $$\begin{array}{c} {\dot{n}}_{res}=P-\frac{n_{res}}{\tau_{r}}-\frac{n_{res}\left(1-f_{4}\right)}{\tau_{c}}+\frac{D_{4}f_{4}}{\tau_{e4}}\\ {\dot{f}}_{4}=-\frac{f_{4}}{\tau_{r}}+\frac{n_{res}\left(1-f_{4}\right)}{D_{4}\tau_{c}}-\frac{f_{4}}{\tau_{e4}}+\frac{D_{3}f_{3}\left(1-f_{4}\right)}{D_{4}\tau_{e3}}-\frac{f_{4}\left(1-f_{3}\right)}{\tau_{0}}-\frac{G_{sat4}}{D_{4}}v_{gr}s_{4}\left(2f_{4}-1\right)\\ {\dot{f}}_{2,3}=-\frac{f_{2,3}}{\tau_{r}}-\frac{f_{2,3}\left(1-f_{3,4}\right)}{\tau_{e2,3}}+\frac{D_{3,4}f_{3,4}\left(1-f_{2,3}\right)}{D_{2,3}\tau_{0}}-\frac{f_{2,3}\left(1-f_{1,2}\right)}{\tau_{0}}+\frac{D_{1,2}f_{3,4}\left(1-f_{2,3}\right)}{D_{2,3}\tau_{e1,2}}-\frac{G_{sat2,3}}{D_{2,3}}v_{gr}s_{2,3}\left(2f_{2,3}-1\right)\\ {\dot{f}}_{1}=-\frac{f_{1}}{\tau_{r}}-\frac{f_{1}\left(1-f_{2}\right)}{\tau_{e1}}+\frac{D_{2}f_{2}\left(1-f_{1}\right)}{D_{1}\tau_{0}}-\frac{G_{sat1}}{D_{1}}v_{gr}s_{1}\left(2f_{1}-1\right)\\ {\dot{s}}_{i}=-\frac{s_{i}}{\tau_{s}}+G_{sat\left(i\right)}v_{gr}s_{i}\left(2f_{i}-1\right)+\beta \frac{f_{i}}{\tau_{r}},\;\;i=1..4 \end{array}$$

Here, $n_{res}$ is the number of free carriers in the reservoir per one QD; $f_{i}$ is the average population of i-th state of QDs (ground-state and first, second and third excited-states; $s_{i}$ are the quantities of photons in respective photon modes divided by $N_{s}/b$; $N_{s}$ is the total surface density of QDs (i.e., surface density in one layer of quantum dots multiplied by the number of layers); b is the total thickness of the reservoir; P is the pumping intensity related to epy injection current density j via the electron charge e as:

(2) $$j=eN_{s}P.$$

$D_{i}$ is the degeneracy of the i-th level of QDs; for simplicity, we treat QD as a spherical, so that $\;D_{i}=2\left(2\left(i-1\right)+1\right)$; $v_{gr}=c/n$ is the group velocity, *c* is a speed of light in vacuum and n=3.5 is GaAs refractive index; the characteristic times are: $\tau_{r}$ for spontaneous recombination, $\tau_{0}$ for relaxation to lower QD electron level, $\tau_{c}$ for an electron capture on the top QD level. These times play the role of fitting parameters for our model, whereas thermal emission times are related to them as:

(3) $$\tau_{ei}=\frac{D_{i}\tau_{0}\exp\Delta E_{i}/kT}{D_{i+1}},$$

where $\Delta E_{i}$ is the transition energy from the previous level, and it can be calculated from the spontaneous emission wavelengths as:

(4) $$\Delta E_{i}=\xi hc\left(\lambda_{i+1}^{-1}-\lambda_{i}^{-1}\right),\;\;\xi \approx 0.5,$$

where ξ accounts to the share of the electron energy (as opposed to the hole) in total photon energy difference.

Photon lifetime has the form of:

(5) $$\tau_{s}=\frac{n}{c\left(\alpha_{out}+\alpha_{in}\right)},$$

where $\alpha_{out}$ is output optical loss dependent on the microdisk diameter and extracted from experimental data as described above, and $\alpha_{in}$ is internal optical loss manifested by free carrier absorption in the reservoir and having the form of:

(6) $$\alpha_{in}=\alpha_{0}+\frac{\sigma n_{res}N_{s}}{b},$$

Here, we suggest that optical mode has unit optical confinement factor in the reservoir; $n_{res}N_{s}/b$ is the volume concentration of free carriers (we have to emphasize that this term is temperature-dependent); free carrier absorption cross-section $\sigma =4\cdot 10^{-17}\;{\mathrm{cm}}^{2}$ [39] (here, an adjustment for a different wavelength has been made assuming $\sigma \sim \lambda^{2}$) and $\alpha_{0}\approx 3.5$ cm^−1^ is the internal loss due to intrinsic carrier absorption or light scattering, as was estimated from the broad-area lasers made of the same epitaxial wafer [27]. The value for saturated gain on the ground level was also estimated from the experiment and found to be as $G_{sat1}=33\;{\mathrm{cm}}^{-1}$. The values of saturated gain on other levels take the form:

(7) $$G_{sat\left(i\right)}=G_{sat1}\frac{D_{i}}{D_{1}},\;\;i=2..4.$$

Finally, coefficient β is the fraction of photons entering the lasing mode from spontaneous emission. We have found that varying it between $10^{-7}..10^{-5}$ has no considerable effect on threshold current density, and it is only needed to ‘seed’ the stimulated emission.

It is worth mentioning that there exists an alternative approach to describe the system, namely considering electrons and holes independently. This work [40] has succeeded in explaining the switch between one-level and two-level lasing, as well as GS lasing quenching, by introducing a single hole level for both ES and GS levels (there, only two electron levels were considered). However, we were not able to replicate the temperature dependences that we see in the experiment using this model.

Finally, there is the following simplification that we have adopted in our model. We assume that free carrier absorption takes place both in wetting layers (quantum wells) and bulk GaAs spacers; however, we do not separate them in our $n_{res}$ population and do not consider carrier exchange between them. The emission time from the top excited level used in our model was calculated with $\lambda_{5}=980\;\mathrm{nm}$, as seen in the spontaneous electroluminescence spectra for the leftmost peak (see Figure 1). The justification for this is the following: suppose we had a separate equation for the population of GaAs matrix. The exchange terms in it will only contain the QW and matrix population and bear the opposite sign to those that are found in the equation for the QW population. By adding the QW and the bulk equation, we will eliminate the exchange terms and retain a single equation for $n_{res}$, which, in this case, will be the sum of QW and GaAs population. The only place where $n_{res}$ is present is the equation for $f_{4}$, the top QD excited-state. However, in exchange terms, there also contain $\tau_{c}$ and $\tau_{e4}$ that is connected to it via Equation (3). So, as a fitting parameter, $\tau_{c}$ accounts for whatever exchange is happening within the reservoir, i.e., between GaAs and QW, while also facilitating keeping the equations succinct.

The Equation (1) can be solved numerically either as differential equations, with the stationary value calculated at sufficiently large value of time (practically: about an order of magnitude longer than the largest τ in the system), and the threshold current calculated as a value of $j_{thr}$, for which the second derivative $s_{1\left(2\right)}^{’’}\left(j\right)$ is maximal, while the first derivative is positive (the latter condition is necessary for discarding possible false values of current where quenching starts). Alternatively, we can put forward two conditions for GS, ES, and two-level lasing thresholds, respectively:

(8) $$\begin{array}{c} s_{i}=0,\;2f_{1}-1=\frac{\alpha_{in}+\alpha_{out}}{G_{sat1}},\;\;\left(\mathrm{GS}\;\mathrm{lasing}\;\mathrm{threshold}\right)\\ s_{i}=0,\;2f_{2}-1=\frac{\alpha_{in}+\alpha_{out}}{G_{sat2}},\;\;\left(\mathrm{ES}\;\mathrm{lasing}\;\mathrm{threshold}\right)\\ s_{2,3,4}=0,\;s_{1}\neq 0,\;2f_{1,2}-1=\frac{\alpha_{in}+\alpha_{out}}{G_{sat1,2}},\;\;\left(\mathrm{GS}+\mathrm{ES}\;\mathrm{lasing}\;\mathrm{threshold}\right) \end{array}$$

and solve Equation (1), replacing all the temporal derivatives with zeros and combining with (8), as algebraic equations. We found out that these two methods yield the same results.

### 3.5. Numerical Modelling Results for Two-State Lasing

**Figure 4:**
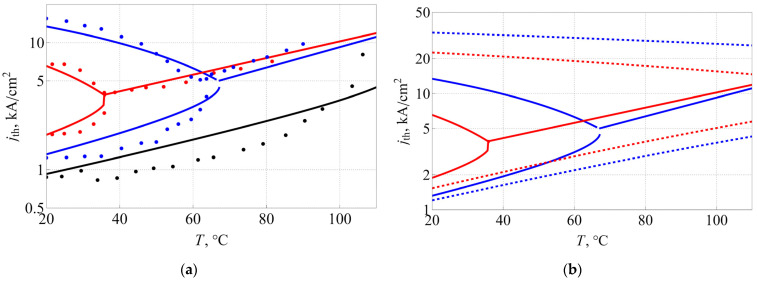
Temperature evolution of the threshold current densities of GS (lower curve until intersection point) and ES (higher curve if present) lasing for microdisks of different diameters (28 μm: black, 24 μm: blue, 20 μm: red). Solid lines denote calculation; dots denote the experimental data (**a**); comparison of the temperature dependences of threshold current densities calculated with (solid lines) and without (dotted lines), taking into account free carrier absorption dependency on reservoir population, as described by Equation (6) (**b**). Colors denote data for microdisks of different diameters (24 μm: blue, 20 μm: red).

By fitting the experimentally obtained temperature dependences of the threshold current densities for the 20, 24 and 28 μm lasers, presented in Figure 3a, (estimated from the results obtained in [27], output loss $\alpha_{out}$ equal to 21 cm^−1^, 18.3 cm^−1^, and 15 cm^−1^, respectively), we were able to extract the following parameters for our model: $\tau_{r}=400\;\mathrm{ps}$, $\tau_{0}=0.55\;\mathrm{ps}$, $\tau_{c}=27\;\mathrm{ps}$. The fitted results are displayed in Figure 4a.

It can be seen that there is a good agreement between the theory and the experiment; most importantly, we were able to replicate the switching between two-state lasing and ES-only lasing at elevated temperatures. This proves that the mechanism that governs this switch consists of two main parts: (I) free carrier absorption, dependent on the reservoir population; and (II) a multilevel system with narrow energy gaps between levels, which facilitates temperature-induced carrier escape from quantum dots.

The importance of considering internal losses as function of temperature is illustrated in Figure 4b, where we compare threshold current densities calculated with our main model, described above, and with a simplified one, where internal loss is constant. It is immediately evident that disregard for loss temperature dependence leads to overestimation of ES lasing threshold and generally to a much smaller slope in temperature dependences of lasing threshold currents. This, in turn, makes the GS–ES lasing intersection point move towards much higher temperatures, which completely contradicts the experimental data. This comparison proves that the main mechanism responsible for GS lasing quenching is the growth of free carrier absorption because of intensification of carrier thermal escape from QDs with elevated temperatures, as well as because of higher effective density of states. This, in turn, leads to higher population of the reservoir and higher free carrier absorption, as per Equation (6). As soon as internal loss exceeds $G_{sat1}$, the GS lasing becomes impossible, and only ES lasing can occur, which is what we witness in experiment and in calculations for higher temperatures.

**Figure 5:**
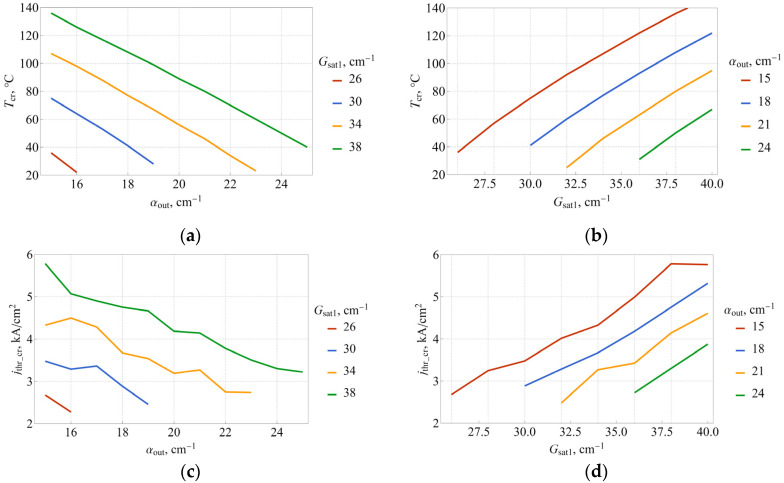
Critical temperature (**a**,**b**) and corresponding threshold current density (**c**,**d**) as a function of $\alpha_{out}$ (**a**,**c**) and $G_{sat1}$ (**b**,**d**).

Since the total loss is the sum of internal and output losses, we see that the critical temperature is lower for higher output loss, i.e., for smaller microdisks. Figure 5a,b show the critical temperature and the respective threshold current density $j_{thr\_cr}$ (that is, coordinates of the intersection point of the curves in Figure 4) as a function of output loss for various values of $G_{sat1}$ and as a function of $G_{sat1}$ for various output losses, respectively. It is clear that temperature dependence is almost linear for a wide range of both variables, whereas threshold current dependences, presented in Figure 5c,d, exhibit some irregularities. Nevertheless, both dependences can be subjected to linear fitting, which yields:

(9) $$\begin{array}{c} T_{cr}=244\;K+8.27G_{sat1}-9.85\alpha_{out}\\ j_{thr\_cr}=-0.2\;{\mathrm{kA}/\mathrm{cm}}^{2}+0.246G_{sat1}-0.244\alpha_{out} \end{array}$$

Here, the variables $G_{sat1}$ and $\alpha_{out}$ should be taken in cm^−1^, as usual. The maximum residuals for the fit are 4 K for the temperature and 0.3 kA/cm^−1^ for threshold current density, suggesting that linear fit is indeed a good approximation. While the applicability of the Equation (9) is naturally limited, it still points out a rather elevated sensitivity of both dependences on saturated gain and output loss, when a change of just 1 cm^−1^ leads to sizable change in critical temperature and current. It should be noted that the coefficients describing the effect of output loss and saturated gain are close but not equal to each other, which is due to the presence of internal loss. Given the relationship between output loss and microlaser diameter, the critical temperature was calculated as a function of diameter. The results presented in Figure 3a show an acceptable agreement between simulation and experiment.

## 4. Conclusions

In the two-state lasing regime, the microdisk laser simultaneously emits several groups of modes falling into different optical transitions of QDs. Spectral separation between them is about 80 nm, which significantly exceeds the distance between neighboring whispering gallery modes. In microdisks with a diameter larger than 20 μm, the ground-state and the first excited-state optical transitions are involved into two-state lasing. However, in a smaller microdisk, lasing already starts on the ES1 optical transition, and then the ES2 laser emission ignites. The current density at which two-state lasing appears decreases with a decrease in the microdisk diameter and/or an increase in temperature. Modelling reveals that temperature affects two-state lasing in two ways. First, a rise in temperature enhances the population of the higher-energy level, and thus the optical gain on the excited-state optical transition, sufficient for lasing, is achieved at a lower injection level. Second, a temperature-induced growth of the carrier concentration results in additional optical loss due to free carrier absorption. Accounting for the latter effect is essential for an adequate description of the temperature characteristics. For example, if a rise of the internal loss is not taken into consideration, the critical temperature, at which GS lasing is still possible, is overestimated by about 100 °C compared to the experimental results. Our experimental results and calculations show that, in order to achieve a two-state lasing regime at elevated temperatures, it is necessary to reduce the output loss (which is not always possible or convenient, since it requires large microlaser diameters) or increase the saturated gain (which can be implemented by increasing the optical confinement factor of the QDs).

## Data Availability

The data presented in this study are available on request from the corresponding author. The data are not publicly available due to the author’s readiness to provide it on request.
